# Suture‐Related Delayed Epidural Hematoma Following Cervical Open‐Door Laminoplasty: A Report of Two Cases and Literature Review

**DOI:** 10.1111/os.70400

**Published:** 2026-08-05

**Authors:** Xiaoxuan Huang, Can Guo, Junbo He, Yi Yang, Kangkang Huang, Hao Liu, Chen Ding

**Affiliations:** ^1^ Department of Orthopedics, Orthopedic Research Institute West China Hospital, Sichuan University Chengdu China; ^2^ Department of Rehabilitation Medicine Center and Institute of Rehabilitation Medicine West China Hospital, Sichuan University Chengdu Sichuan China; ^3^ Key Laboratory of Rehabilitation Medicine in Sichuan Province Chengdu Sichuan China

**Keywords:** barbed suture, cervical laminoplasty, delayed postoperative spinal epidural hematoma (DPSEH), intramuscular hematoma, suture complication

## Abstract

**Background:**

Delayed postoperative spinal epidural hematoma (DPSEH) is a rare but serious complication of cervical open‐door laminoplasty, which occurs more than 3 days after the procedure. Although multi‐level surgery, coagulopathy, and hypertension are known risk factors, suture‐related mechanisms have rarely been identified as the main cause.

**Case Presentation:**

We present two cases of symptomatic DPSEH occurring on the seventh postoperative day following posterior cervical open‐door laminoplasty. Following initial recovery, both patients experienced acute neurological deterioration. An emergency MRI revealed hematomas within the paraspinal muscles compressing the spinal cord. Intraoperative exploration identified a huge hematoma within the posterior cervical muscles and active bleeding from muscle tissue, with no injury to the epidural venous plexus. Interestingly, absorbable barbed sutures were used for muscular and fascial closure in both procedures. The patients exhibited significant neurological recovery following emergency hematoma evacuation.

**Conclusion:**

These cases suggest that suture‐related complications, specifically knot loosening, suture migration, or barb‐induced tissue cutting, may contribute to intramuscular bleeding and subsequent DPSEH. However, given the limited evidence from a two‐case report, this association should be interpreted as a clinical alert rather than definitive proof of causality. Surgeons should maintain a high index of suspicion for DPSEH in patients who experience sudden postoperative pain or neurological decline. They should also consider suture choice as a potentially modifiable risk factor.

## Graphical abstract

We report two cases of delayed postoperative spinal epidural hematoma occurring on postoperative Day 7 after cervical open‐door laminoplasty. Emergency MRI showed large paraspinal intramuscular hematomas compressing the cord, and re‐exploration found loose barbed sutures with muscle bleeding. Urgent evacuation led to marked neurological recovery, highlighting suture choice as a modifiable risk factor.

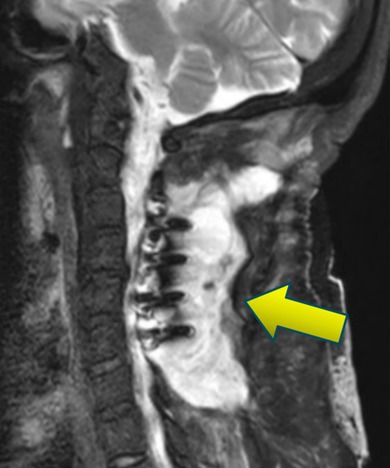

## 1 Introduction

Epidural hematoma (EH) formation after spinal surgery, despite being rare (occurring in 0.1%–0.5% of cases), is a serious complication that can result in symptomatic compression of the spinal cord. Such postoperative hematomas have the potential to seriously harm the nervous system. However, it is extremely uncommon for spontaneous spinal epidural hematomas (SSEH) to develop more than 3 days following posterior cervical open‐door laminoplasty. Here, we report two patients who had unilateral open‐door‐type laminoplasty and experienced a rare case of delayed postoperative spinal epidural hematoma (DPSEH).

First introduced by Hirabayashi et al. in 1977, the open‐door laminoplasty (LP) remains a standard and effective technique for posterior cervical decompression, achieving adequate canal expansion while preserving spinal motion [1]. For patients complicated with Ossification of the Posterior Longitudinal Ligament (OPLL), LP demonstrates superior efficacy. Research indicates that the rate of neurological improvement after LP can reach 91.6% [2].

Nevertheless, some postoperative complications may still arise despite LP's notable clinical efficacy in treating CSM and OPLL. The most common postoperative complication is cervical kyphotic deformity [3]. C5 nerve root palsy is a relatively common complication following posterior open‐door laminoplasty of the cervical spine, which may be associated with traction or compression of the nerve root during the surgical procedure [4]. One possible side effect of open‐door laminoplasty is displacement of the cervical plate, a crucial implant used to stabilize the elevated lamina [5]. Another uncommon side effect after LP is the development of a postoperative EH. Although it is unusual, patients may experience potentially fatal quadriplegia within hours. Therefore, during preoperative assessment, special attention must be paid to risk factors for EH formation to reduce the risk of postoperative complications.

## 2 Case Presentation

### 2.1 Case 1

A 43‐year‐old man presented to our department and reported a 3‐year history of cervicoscapular pain. Both hands were numb, and the pain had gotten worse over the previous 3 months. During the admission physical examination, we obtained a blood pressure reading of 131/92 mmHg. During the neurological examination, we observed hyperesthesia of superficial sensation in the left index and ring fingers compared with the right, exaggerated deep tendon reflexes, and bilateral handgrip weakness.

**FIGURE 1:**
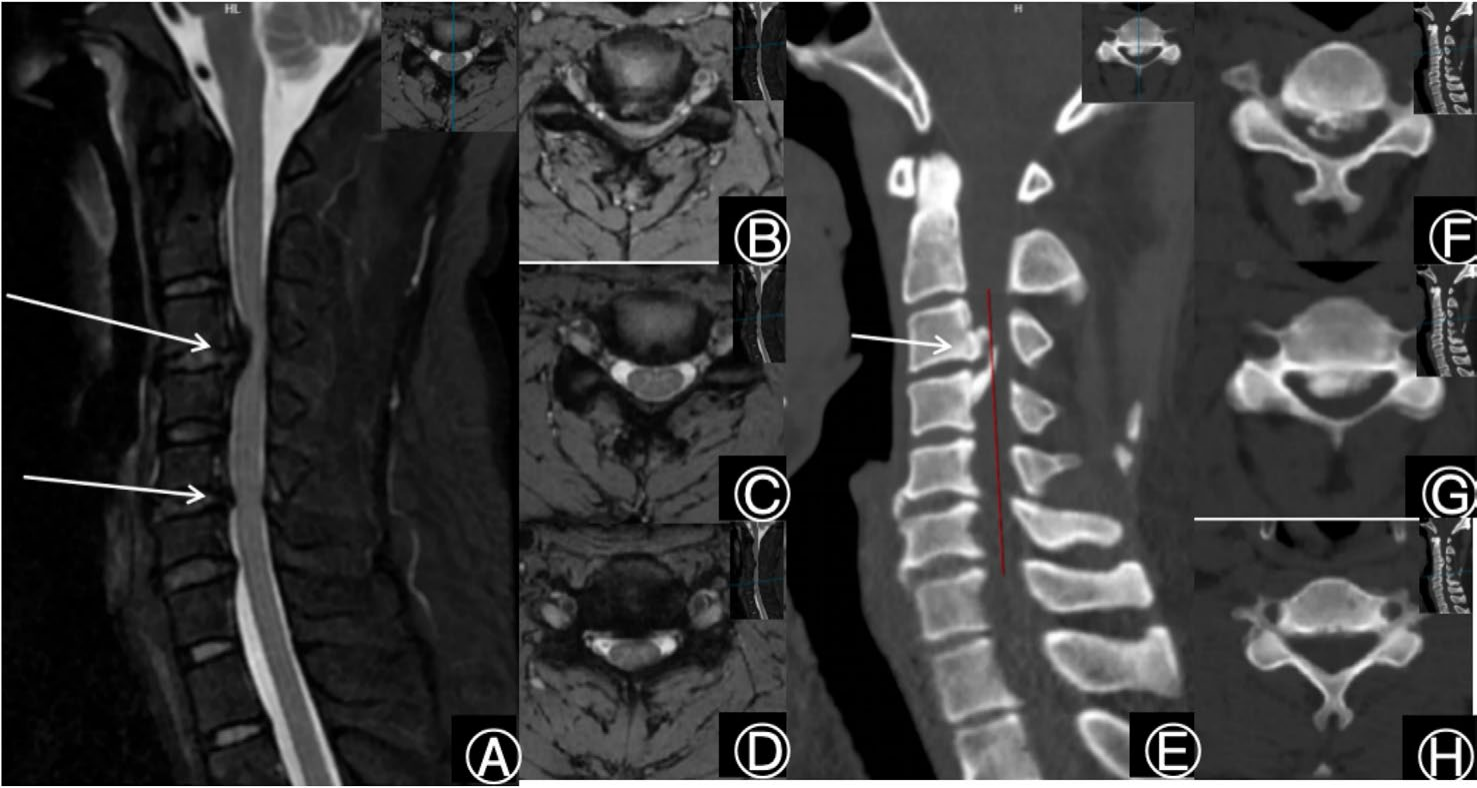
(A) Sagittal cervical MRI T2 view demonstrates multi‐level stenosis from C3 to C7. The axial view revealed the spinal canal being occupied by the ossific ligaments, along with lateral recess stenosis (B–D arrow). Cervical spine CT scan revealed OPLL posterior to C3 and C4 vertebrae. (E) Sagittal reconstruction CT. (F–H) Axial CT cuts of C3_C5. K‐line is negative (E, red line). OPLL posterior to the C3 and C4 vertebrae, with a canal occupying ratio of approximately 60% (F, G).

With bilateral C3/4 neural foraminal stenosis and an approximate spinal canal occupancy rate of 60%, computed tomography (CT) reveals ossification of the posterior longitudinal ligament (OPLL) posterior to C3 and C4 vertebrae. The K‐line is negative (Figure 1E–H). Magnetic resonance imaging (MRI) demonstrated C3/4 central disc extrusion causing thecal sac and spinal cord compression, accompanied by cord thinning and edema. Additional disc bulging was observed at C5‐7 (Figure 1A–D).

**FIGURE 2:**
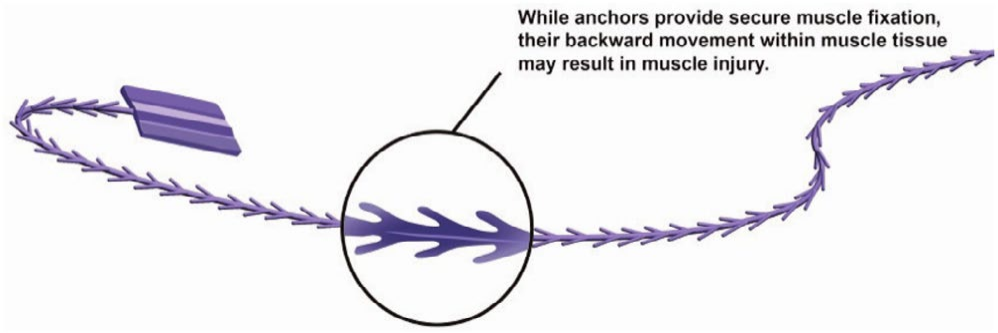
The details of barbed suture.

**FIGURE 3:**
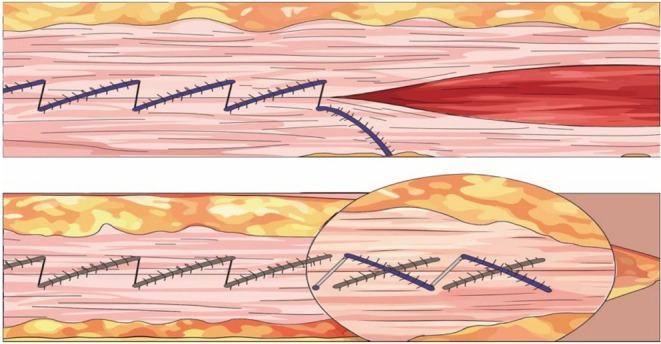
A schematic illustration to show the suture technique. Suture loosening, migration, or barb‐induced cutting may lead to focal paraspinal muscle bleeding.

**FIGURE 4:**
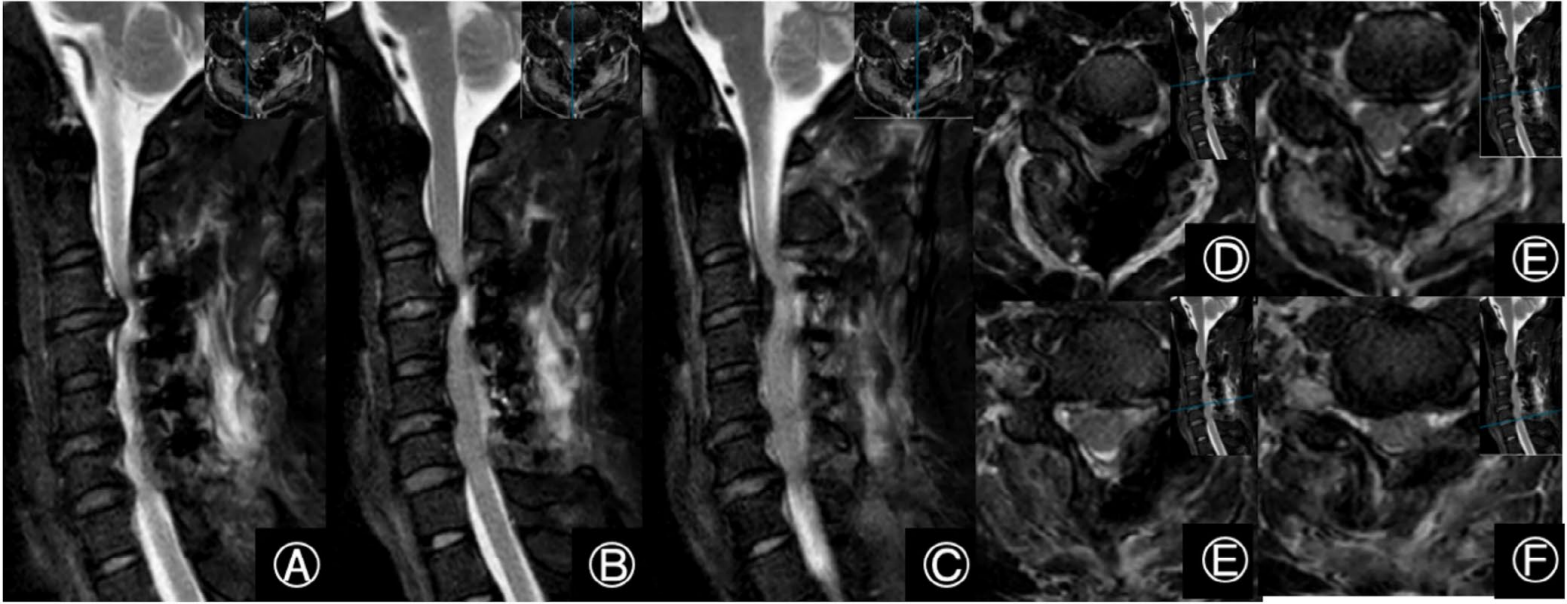
Cervical MRI on postoperative Day 3. The follow‐up imaging displayed that the spinal cord compression was partially relieved, with inadequate posterior migration observed at C3 and C4 levels.

**FIGURE 5:**
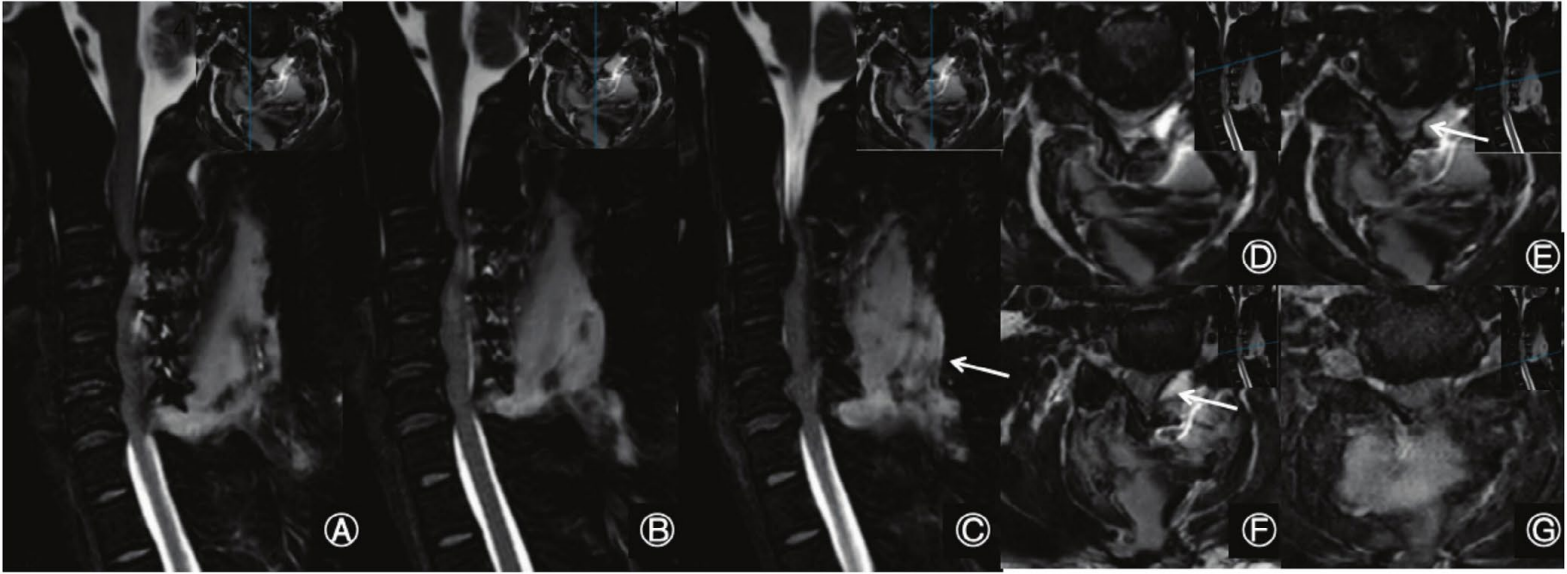
An emergent MRI on postoperative Day 7 revealed a large hematoma within the paraspinal muscles posterior to the C2–C5 laminae, compressing the spinal cord.

The patient had a cervical laminoplasty from C3 to C6, which was completed with little bleeding and no side effects. During wound closure, the posterior cervical paraspinal muscle and deep fascial layers were closed using a 2–0 absorbable barbed suture (STRATAFIX, Ethicon, Johnson & Johnson, USA). The suture was placed in a continuous running fashion from the caudal to the cranial end of the incision, with each bite incorporating adequate musculofascial tissue to ensure layer‐by‐layer approximation and uniform tension distribution(Figures 2 and 3). At the end of the closure, several reverse bites were placed to minimize the risk of suture slippage, and the terminal end was secured in accordance with the manufacturer's recommended technique. The entire closure procedure was performed according to the manufacturer's instructions for use and the technique described in the official barbed suture brochure. On the third postoperative day, an MRI confirmed that the compression of the spinal cord had somewhat subsided (Figure 4). The endotracheal tube was successfully removed on the third postoperative day. On the seventh postoperative day, at around 1:30 AM, the patient reported pain at the cervical wound site and experienced a sudden onset of impaired movement in all four limbs. Physical examination demonstrated grade 0 muscle strength in all four limbs, accompanied by decreased muscle tone, absent deep tendon reflexes, and progressively decreasing blood pressure. With suspicion of an EH, an emergency 3D CT of the cervical spine and a metal artifact reduction MRI of the cervical spine were performed. A sizable hematoma within the muscles at the C5/6 level compressing the spinal cord was visible on the MRI (Figure 5). The patient underwent an emergency posterior approach surgery at 8:00 AM for cervical hematoma exploration and evacuation. The procedure involved partial laminectomies at C2 and C7, a complete laminectomy at C3, and the placement of a drainage tube. During the procedure, 100 and 150 mL of dark, clotted blood were removed through the posterior cervical incision, compressing the thecal sac. The patient's sensory function improved significantly in all four limbs after surgery.

In the subsequent months, the patient's muscle strength recovered gradually from grade 0 to grade 4. Concurrently, pathological reflexes and deep tendon function returned to normal.

### 2.2 Case 2

**FIGURE 6:**
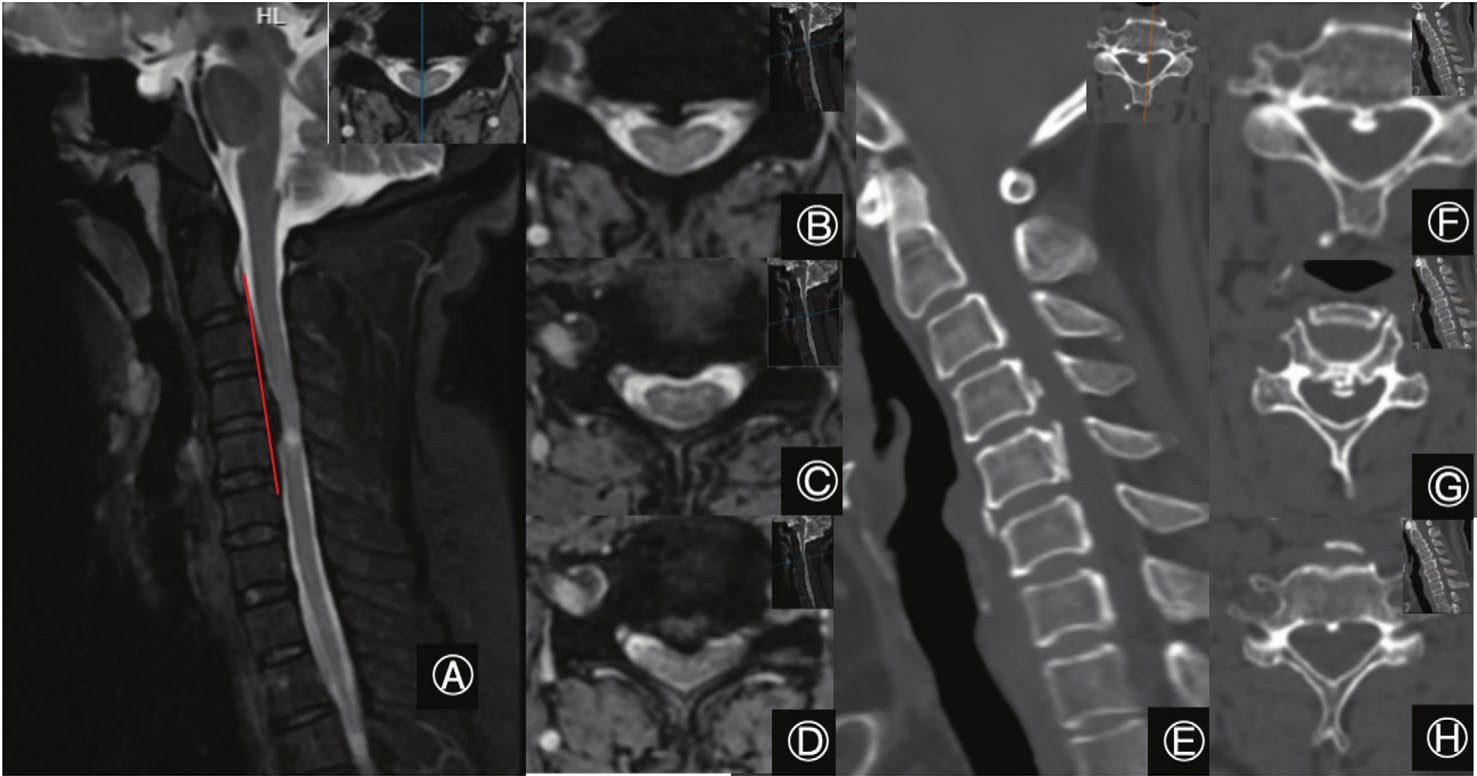
Case 2 Preoperative magnetic resonance imaging demonstrated: A negative K‐line and spinal cord compression at C3–C7 levels on coronal views.

A 54‐year‐old woman experiencing cervicoscapular pain for 5 months was admitted. The pain has gotten worse for 1 month. She complained of abnormalities similar to electric shocks in all four limbs, urinary dysfunction, and an unsteady gait. Her past medical history reported no smoking use. On admission, a blood pressure of 140/90 mmHg was recorded. Reduced feeling in the right ring finger, grade 4/5 bilateral hand grip strength, lower limb hyperreflexia, and positive pathological signs were found during the examination. Preoperative MRI revealed C3–C7 disc herniations, dural sac compression, and spinal stenosis. Mild OPLL posterior to C3 and C7 vertebrae was seen on CT (Figure 6).

The patient underwent posterior cervical C3‐C6 open‐door laminoplasty with centerpiece fixation under general anesthesia. The fascia and paraspinal muscle layers were sutured using 2–0 absorbable barbed sutures, as in Case 1. Before wound closure, a drainage tube was inserted. The procedure went normally, with approximately 200 mL of blood loss.

**FIGURE 7:**
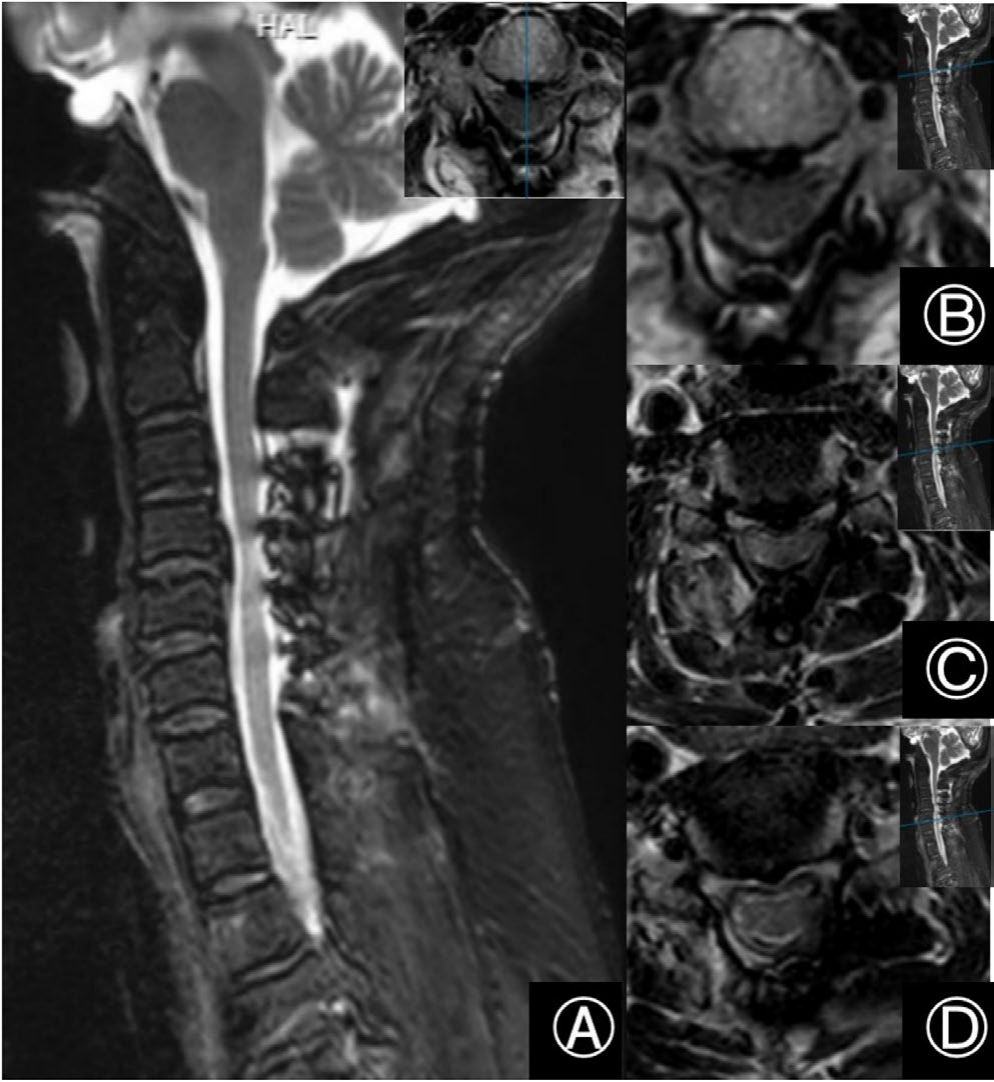
The follow‐up MRI on postoperative Day 3 revealed spinal canal expansion with posterior migration of the spinal cord, absence of cord compression, and no significant hematoma posterior to the vertebral bodies or within the spinal canal.

On the fifth postoperative day, the follow‐up MRI (Figure 7) demonstrated spinal canal expansion and resolution of spinal cord compression. The following day, the drain was removed, and the patient began ambulation while wearing a cervical collar.

**FIGURE 8:**
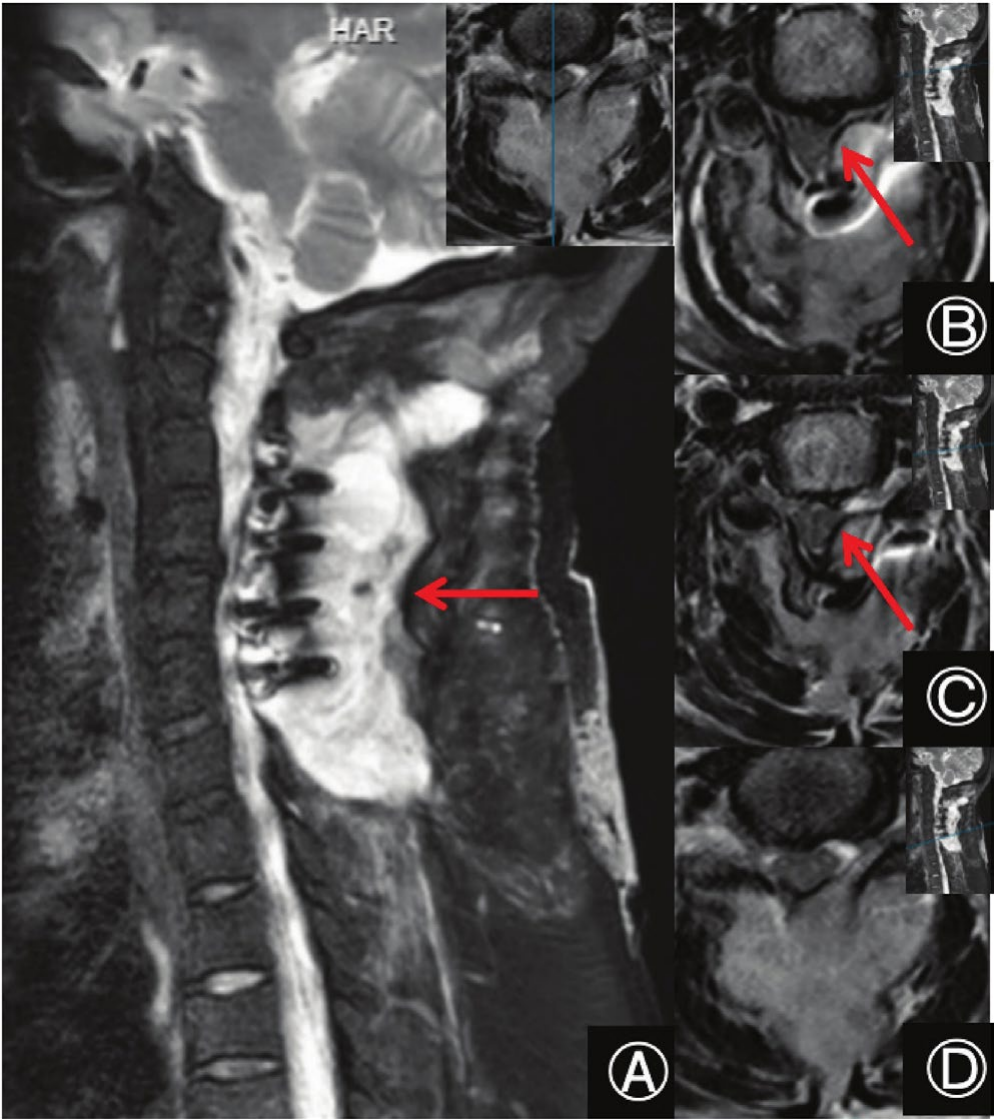
An emergency MRI performed on postoperative Day 7 following hematoma formation revealed a large retrovertebral hematoma, which caused spinal canal stenosis and cord compression at C4–C6 levels.

**FIGURE 9:**
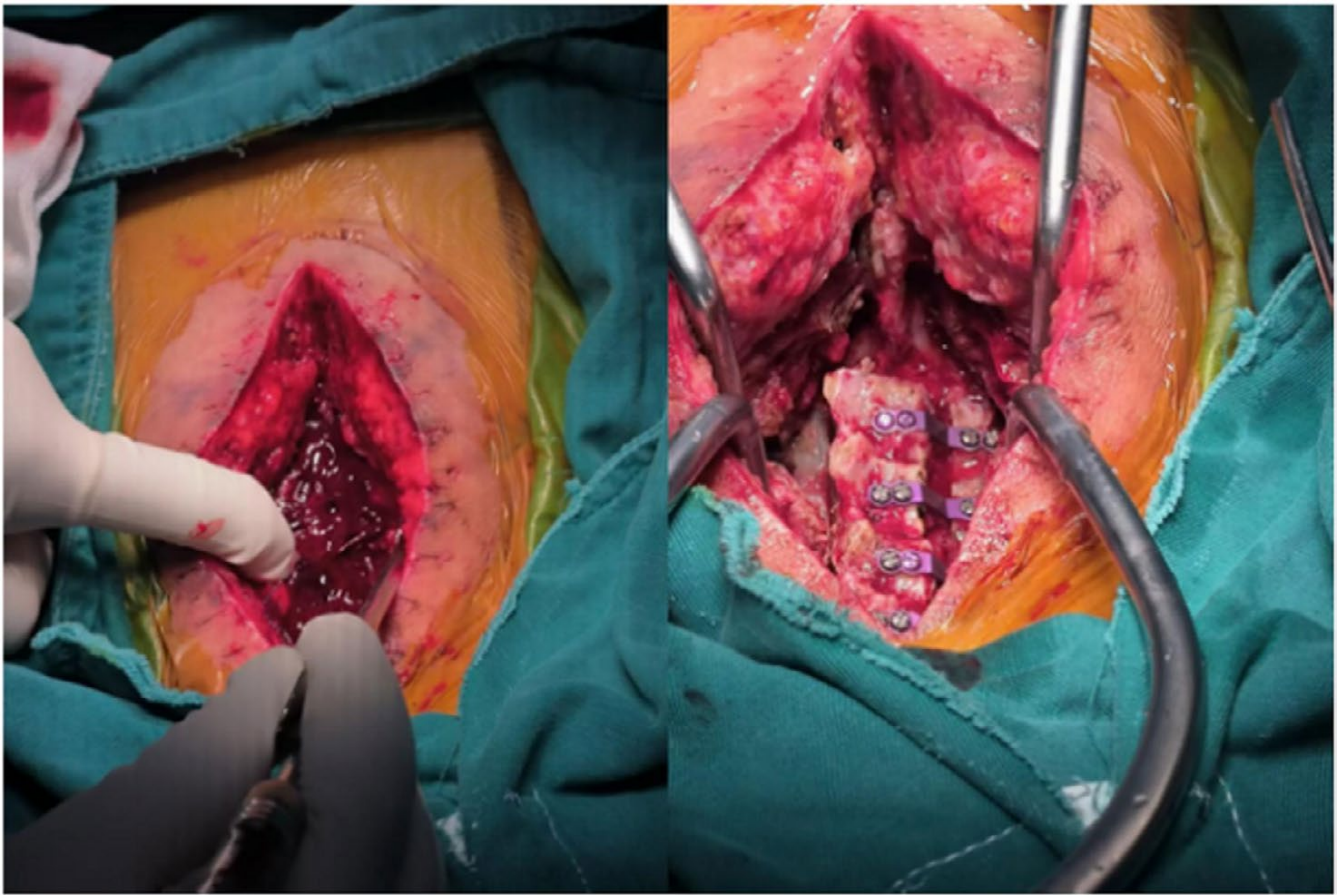
The left image displays the paraspinal muscles and the posterior to the spinal canal. The right image displays the plate and screw fixation, which remained secure with no active bleeding observed from the bone resection sites after evacuation of the hematoma.

The patient unexpectedly developed excruciating pain at the wound site on the seventh postoperative day. Emergency MRI (Figure 8) suggested the possibility of a delayed hematoma following posterior cervical surgery. Emergency posterior cervical hematoma exploration and evacuation, spinal canal decompression, and drainage tube implantation were carried out. During surgery, blood clots were found within the posterior cervical muscles and in the epidural space, but no active bleeding site was identified (Figure 9). Postoperatively, the patient reported improvement in neck pain, and physical examination demonstrated normal muscle strength and tone in all four limbs.

## 3 Discussion

### 3.1 EP Incidence

One frequent surgical complication is a postoperative spinal EH. Using MRI, Mirzai et al. found that the incidence of postoperative EH can be as high as 89% [6]. On the other hand, symptomatic postoperative EH is uncommon, occurring in 0.1%–3.0% [3, 7, 8, 9, 10, 11, 12, 13]. Additionally, there are differences in the incidence of symptomatic hematomas between lumbar and cervical surgery. This could be due to the larger cross‐sectional area of the lumbar canal and greater pressure tolerance of nerve roots. The most recent postoperative spinal EH was reported to have started on the 20th postoperative day [14]. Nevertheless, the incidence of delayed hematoma formation after cervical open‐door laminoplasty is even lower, with only rare case reports published to date. Furthermore, in some reports of DPSEH, the EH might have been present immediately after surgery but remained asymptomatic, only manifesting neurological symptoms and being diagnosed as a delayed hematoma more than 3 days postoperatively.

### 3.2 Risk Factors

Previous research has identified risk factors for developing an EH within 3 days following spinal surgery, including the use of non‐steroidal anti‐inflammatory drugs (NSAIDs) before or after surgery, advanced age (> 60 years), Rh‐negative blood type, hypertension, and failure to perform postoperative drainage. Preoperative coagulation disorders and multi‐level surgery (more than five surgical levels) have been identified as risk factors for developing postoperative EH [15, 16, 17]. Preoperative NSAID use and significant intraoperative blood loss have also been found to be risk factors [17, 18]. Delayed EH has been linked to several causes, according to the literature currently in publication (Table 1). Uribe et al. suggested that the incidence of DPOSEH is higher in patients who have had previous spinal surgery at the same level [11]. Even in young adults in good health, a seemingly insignificant incident, such as coughing, can put stress on the surgical wound and cause hematoma formation up to 10 days after the procedure, even after the sutures have been removed and the wound has healed [19]. There is ongoing debate regarding the effect of drain placement on the probability of EH formation [7, 20]. The epidural venous plexus is typically thought to be the source of SEH, and disc herniation may rupture Batson's epidural veins and cause epidural coagulation [10, 11, 21]. Reported risk factors associated with delayed EH include multi‐level surgery, prolonged surgical duration, significant intraoperative blood loss, preoperative coagulation disorders, and concomitant OPLL. Identified as an important risk factor, the presence of OPLL may be closely linked to an increased number of surgical segments [22, 23]. Moreover, patients undergoing posterior approach surgery that involves two or more segments have a significantly higher complication rate in comparison to those receiving single‐level procedures. This disparity is most probably due to the greater technical complexity and longer operating times of multi‐level surgeries [24].

**TABLE 1:** Literature review of published cases of delayed postoperative spinal epidural hematoma.

Author	Basic information	Surgical procedure	Time of hematoma onset	Symptoms	Causes
Neo	59, M	French door–type C3–T1 laminoplasty	Postoperative Day 9	Severe neck and shoulder pain, then tetraplegia rapidly developed	Caused by straining while attempting to defecate
Tomii	56, M	Open door‐type C4–C6 laminoplasty	Postoperative Day 7	Suddenly felt severe stabbing neck and shoulder pain, then tetraplegia rapidly developed	Continuous hypertension
Khan	55, M	C3‐7 laminectomy and instrumented fusion with lateral‐mass screws and rods	Postoperative Day 9	Progressively increasing severe neck pain and muscle spasms with mild paresthesias radiating down to the fingertips	Progressively increasing severe neck pain and muscle spasms with mild paresthesias radiating down to the fingertips
Xu	85, M	C3/4 and C4/5 ACDF	Postoperative Day 5	Limb weakness accompanied by dysuria, with Grade 2 muscle strength	Old age, RH‐positive blood group, hypertension, smoking, and low platelet count before the operation, and multi‐stage operation
Sokolowski	82, F	L2 to S1 decompressive laminectomy and uninstrumented posterolateral fusion with autograft	Postoperative Day 6	A lack of bowel control and new onset of left lower extremity pain	Unknown
Sokolowski	86, F	L2 to L5 laminectomies and L2 to S1 uninstrumented posterolateral spinal fusion with autograft and allograft bone	Postoperative Day 13	Significantly increased level of pain and decreased strength in her bilateral lower extremities	A small bleeding rim of bone was noted at the proximal extent of the decompression
Uribe	55, F	Cervical laminoplasty	Postoperative Day 6	A sharp, severe pain at the level of the previous surgery, followed by radicular symptoms consisting of dysesthesias	Unknown
Uribe	52, M	Cervical laminoplasty	Postoperative Day 3	A sharp, severe pain at the level of the previous surgery, followed by radicular symptoms consisting of dysesthesias	Initiation of heparin‐based anticoagulants occurs on postoperative Day 3
Uribe	53, M	ACDF C7–T1 C5–T3 laminectomy and fusion	Postoperative Day 3	A sharp, severe pain at the level of the previous surgery, followed by radicular symptoms consisting of dysesthesias	Postoperative subcutaneous heparin administration Previous spinal surgery at the same level
Uribe	69, F	Cervical laminectomy for recurrent meningioma	Postoperative Day 8	A sharp, severe pain at the level of the previous surgery, followed by radicular symptoms consisting of dysesthesias	Postoperative subcutaneous heparin administration Previous spinal surgery at the same level
Sreenivasan	38, M	An open door type C4‐D1 laminoplasty	Postoperative Day 10	Power in all four limbs deteriorated from 4/5 to 1/5 for 2 h	A bout of cough
David	80, F	A posterior L3–S1 laminectomy and instrumented bilateral lateral fusion of L3–S1	Postoperative Day 16	Bilateral lower extremity numbness and progressive weakness	Anticoagulation with heparin
Zhou FeiFei	78, M	Open door‐type C3 to C7 laminoplasty	Postoperative Day 3	Progressive decline in the strength of both upper limbs	Unknown

A patient with a history of constipation was reported by NEO et al. [25]. It offers a viewpoint on the potential for intramuscular hematomas to cause spinal cord compression, even though suture problems were not discussed. According to Ubribe et al., a major risk factor for the development of DPSEH is prior surgical history [11]. Five PSEH cases (43%) in Anno's study had a delayed onset, highlighting the necessity of careful postoperative monitoring and educating patients and medical personnel about the potential for DPSEH to guarantee prompt intervention [8]. David E. Spanier described a case where an EH was diagnosed after initiating heparin therapy [26]. Given the lack of a clearly defined “safe period” following spinal surgery, it is advisable to consider the use of a vena cava filter for pulmonary embolism prophylaxis. Zhou et al. reported a patient with a history of self‐administered use of aspirin for several years, which was not disclosed to the treating physician [27]. There is currently no recognized and trustworthy window for the safe start of anticoagulant therapy following surgery, as previously noted by Spanier et al. [26]. Moreover, Mark et al. have demonstrated that even when anticoagulant medications are discontinued several weeks before surgery, the patients with history of such medications should be closely monitored by medical staff for the potential development of EH, striving to detect and manage it at an early stage [14]. Additionally, a case reported by Sreenivasan et al. suggests that straining events such as coughing, sneezing, or vigorous neck movements can result in delayed postoperative hematoma [19].

### 3.3 Mechanism Hypothesis

MRI findings in our two reported cases suggest that the bleeding originated from the posterior paraspinal muscles. Therefore, the hypothesis proposed by Gundry et al. cannot explain the pathogenesis of the DPSEH in our cases. Preoperative coagulation function was normal in both patients: platelet counts were > 100 × 10 [9]/L, and APTT, PT, D‐dimer, and fibrinogen were all within normal ranges. As initial symptoms, the first patient developed neck pain, acute neurological symptoms, and quadriplegia on the seventh postoperative day, and the second patient suffered from severe neck pain.

Nevertheless, there have been no documented instances of delayed hematoma resulting in acute cervical spinal cord compression among the several hundred open‐door cervical procedures carried out at our hospital in recent years. Both patients with delayed hematoma formation had a history of ambulation before symptoms appeared, according to a thorough examination of their medical histories. Both patients had follow‐up MRIs on the third and fifth postoperative days, and the MRIs detected no hematoma. We concluded that ambulation was most likely the cause of the hematomas in these two patients after combining this with the emergency MRI that was done following the onset of quadriplegia, which revealed hemorrhage within the posterior cervical muscles leading to hematoma formation in both cases. Although no obvious active bleeding point was identified, during hematoma evacuation for both patients, exploration revealed loose knots in the sutures deep within the muscle layers. We also observed that the barbed suture had retracted within the paraspinal muscle layer, and that the backward movement of the suture allowed the barbs to cut through the muscle tissue, resulting in muscular laceration. A large amount of clotted blood was removed from between the paraspinal muscles. There was no bleeding, and the epidural venous plexus was unharmed. Our conclusion is supported by the fact that this is different from the frequently mentioned cause of spontaneous EH formation. It is therefore reasonable to speculate that after ambulation, due to suture loosening or suture cutting through the tissue, deep muscle re‐bleeding occurred. Postoperative mechanical strain associated with early ambulation, combined with possible suture‐related tissue injury, may contribute to delayed bleeding in vulnerable wounds [25].

Knotless antibacterial barbed sutures consist of an absorbable material with densely arranged barbed structures. Compared to conventional monofilament sutures, knotless barbed sutures offer advantages such as faster suturing speed and less tissue irritation. However, in mobile and high‐tension tissues, repeated cyclic loading may permit limited suture migration or local tissue cut‐through, particularly if the suture is placed through friable muscle fibers or if excessive traction is applied during closure. Because of the barbed structure, if the suture gets tangled or the surgeon uses too much force when suturing, it has a greater chance of getting cut or breaking. Recalling the surgical process, about 6 months ago, we started using knotless barbed sutures for deep tissue, such as muscles. After continuous suturing of the deep fascia, part of the incision was sutured retrograde to prevent suture slippage, followed by knot tying. These two cases were possibly related to the change in suture material. The particular barbed suture used might be prone to retraction, which led to an insufficient approximation of the muscle layers. This could prevent effective compression of the muscle wound, thereby increasing the risk of re‐bleeding from small vessels within the muscle. Due to the nature of the barbed suture, during postoperative ambulation and muscle contraction, the suture can migrate within the muscle, and the barbs could cause cutting injuries to the paraspinal muscles, leading to bleeding from intramural arteries. Additionally, it is challenging to achieve effective tamponade during open‐door laminoplasty because of the relatively large posterior cervical incision, which causes the hematoma to gradually expand. Barbed sutures have worse mechanical qualities than traditional sutures. The paraspinal muscles are exposed to repeated traction during neck extension, flexion, rotation, coughing, and early mobilization. Also, after open‐door laminoplasty, the open side of the lamina may provide a potential route through which a posterior paraspinal hematoma can secondarily compress the dural sac. Compared to other parts of the body, the cervical joint and muscles are subjected to greater stress during spine surgeries. Accordingly, barbed sutures have a higher risk of breaking. A systematic review and meta‐analysis also reported a higher incidence of suture breakage [28]. Recent studies on posterior cervical surgery have emphasized the importance of posterior cervical muscle condition and soft‐tissue preservation [29, 30]. Meticulous muscular closure and careful selection of suture material may be particularly important in posterior cervical surgery. Additionally, Shermak et al. noted that barbed sutures were associated with higher complication rates and challenges in knee extensor mechanism healing, particularly in the context of fascial and subcutaneous tissue closure. Adhesion, inflammation, seroma, erythematous, swollen, gingivostomatitis, depression, incisional infection, and dehiscence are just a few of the numerous side effects of barbed sutures that Xiang et al. reported [31].

### 3.4 Preventive Strategies

When closing posterior cervical wounds, surgeons should tailor the closure strategy to the depth of the incision, soft‐tissue quality, and local tension. Although barbed sutures can shorten wound‐closure time and provide continuous tissue approximation, their use in the deep muscular and fascial layers of the posterior cervical spine requires caution because these layers experience repeated traction during neck motion and early ambulation. In patients with high‐tension paraspinal soft tissue, we suggest reinforcing the deep fascial closure with an additional back‐running pass when this can be achieved without substantially increasing the economic burden; this may enhance the mechanical strength of the deep fascial layer and reduce the risk of wound dehiscence. During continuous suturing, the surgeon should avoid excessive traction and ensure that each bite incorporates adequate healthy musculofascial tissue, rather than only superficial or friable muscle fibers. Careful hemostasis at the paraspinal muscle bed, laminar trough, and bone‐cutting surfaces remains essential before closure. After surgery, particular attention should be paid to the period after drain removal and early ambulation. New‐onset severe wound pain, wound swelling, abnormal drainage, or neurological deterioration after cervical laminoplasty should prompt urgent MRI evaluation and early surgical intervention when hematoma compression is suspected.

### 3.5 Limitations

This study has several limitations. The most important one is the small number of cases. Although both patients showed intraoperative findings suggestive of suture retraction, barb‐related muscle injury, and delayed paraspinal muscle bleeding, two cases cannot prove a causal relationship between barbed sutures and delayed postoperative spinal epidural hematoma. This two‐case series does not permit a causal conclusion. Instead, we issue a clinical alert that surgeons should be aware of the possible association between barbed suture and DPSEH. We also did not perform biomechanical testing, histological assessment, or animal experiments to confirm whether suture migration or barb‐induced cutting directly injured intramuscular vessels. In addition, because there was no control group treated with conventional sutures, we cannot determine whether barbed sutures carry a higher risk of delayed hematoma than standard closure methods. These cases should therefore be interpreted as a clinical warning rather than definitive evidence. Further experimental work and larger clinical studies are needed to clarify how barbed sutures behave in posterior cervical musculofascial closure and to identify the patients or wound conditions in which their use may be less suitable.

Future studies should include larger controlled clinical series comparing barbed and conventional sutures in posterior cervical wound closure. Biomechanical studies using cadaveric or animal paraspinal muscle models are also needed to evaluate the tensile strength, pull‐out resistance, migration behavior, and tissue cut‐through characteristics of barbed sutures under cyclic loading. In addition, animal studies with histological assessment may help clarify whether barb‐related microtrauma can injure intramuscular vessels or impair deep soft‐tissue healing.

## 4 Conclusion

It is crucial to recognize that one of the first signs of EH is an abrupt onset of intense, sharp pain. Neurological deficits may develop minutes to days after the initial pain episode. Acute stretching of the dura and nerve roots by the hematoma is frequently indicated by sharp, throbbing, or “tension‐like” pain at the wound site [32]. To improve neurological recovery once symptoms appear, quick diagnosis and surgical intervention are essential [18, 33]. Even though DPSEH following cervical surgery is rare, postoperative patients should be quickly diagnosed and treated if they experience sudden, sharp wound pain, wound fullness or oozing, or even sensory and motor dysfunction in the limbs. Permanent paralysis becomes possible if the compression lasts longer than 24 h. Conversely, if compression is alleviated within 8 h, recovery is normally expected [26, 34].

We report two cases of delayed symptomatic EH that occurred on the seventh postoperative day following posterior cervical open‐door laminoplasty. The bleeding within the paraspinal muscles that resulted in the hematomas may be associated with loose sutures or sutures that cut through the tissue, but a causal relationship cannot be established from these cases alone. In cervical spine surgery involving areas with high tension and significant mobility, the use of barbed sutures may require particular caution to avoid complications such as suture locking or breakage.

This is the first report of delayed EH associated specifically with suture‐related issues. Concurrently, surgeons would typically consider the possibility of an EH in the acute postoperative period when patients present with symptoms such as limb paresthesia, paralysis, or sharp wound pain.

## Author Contributions

**Can Guo:** software, validation. **Xiaoxuan Huang:** conceptualization, methodology, writing – original draft. **Yi Yang:** formal analysis, investigation. **Hao Liu:** supervision, project administration. **Junbo He:** writing – review and editing, supervision, data curation. **Chen Ding:** supervision, resources, project administration, investigation, funding acquisition. **Kangkang Huang:** validation, visualization, supervision.

## Funding

This work was supported by the Sichuan Science and Technology Program (Grant Nos. 2024YFHZ0163 and 2026NSFSC1570).

## Disclosure

The manuscript submitted does not contain information about medical device(s)/drug(s). The authors declare that all authors listed meet the authorship criteria according to the latest guidelines of the International Committee of Medical Journal Editors, and that all authors are in agreement with the manuscript.

## Ethics Statement

This study was approved by the Ethics Committee of West China Hospital of Sichuan University (Approval No. 2019YFG599). Written informed consent for publication of this case report and any accompanying images was obtained from the patient and the patient's family.

## Consent

All authors provide their consent for publication.

## Conflicts of Interest

The authors declare no conflicts of interest.

## Data Availability

The data that support the findings of this study are available from the corresponding author upon reasonable request.
